# Elevated cerebrospinal fluid glucose levels and diabetes mellitus are associated with activation of the neurotoxic polyol pathway

**DOI:** 10.1007/s00125-022-05693-7

**Published:** 2022-04-05

**Authors:** Celien Tigchelaar, Mark L. van Zuylen, Abraham H. Hulst, Benedikt Preckel, André P. van Beek, Ido P. Kema, Jeroen Hermanides, Anthony R. Absalom

**Affiliations:** 1 Department of Anaesthesiology, University Medical Center Groningen, University of Groningen, Groningen, the Netherlands; 2 Department of Anaesthesiology, Amsterdam UMC – Location AMC, University of Amsterdam, Amsterdam, the Netherlands; 3 Department of Intensive Care, Royal Melbourne Hospital, University of Melbourne, Melbourne, VIC Australia; 4 Department of Endocrinology, University Medical Center Groningen, University of Groningen, Groningen, the Netherlands; 5 Department of Laboratory Medicine, University Medical Center Groningen, University of Groningen, Groningen, the Netherlands

**Keywords:** Cerebrospinal fluid, Diabetes mellitus, Fructose, Hyperglycaemia, Neurocognitive function, Polyol pathway, Sorbitol

## Abstract

**Aims/hypothesis:**

During hyperglycaemia, some glucose bypasses glycolysis and is metabolised via the potentially neurotoxic polyol pathway, in which glucose is metabolised to sorbitol and fructose. Increased polyol concentrations have been demonstrated in the cerebrospinal fluid (CSF) of neurological patients with and without diabetes mellitus. However, polyol levels in patients without evident neurological abnormalities have not been investigated so far. The aim of this study was to determine CSF polyol concentrations in patients without major neurological disease with normal or elevated CSF glucose concentrations.

**Methods:**

This observational cohort study used CSF and plasma analyses, as well as clinical data, from 30 participants of the Anaesthetic Biobank of Cerebrospinal Fluid study. Biomaterial was collected from adult patients scheduled for elective surgery under spinal anaesthesia. CSF polyol concentrations were measured by GC/flame ionisation detector in ten patients with normal CSF glucose levels (group 1), ten patients with elevated CSF glucose levels (group 2) and ten patients with elevated CSF glucose levels and type 2 diabetes (group 3). We compared the concentrations of plasma glucose, CSF glucose, sorbitol and fructose, and CSF polyol/glucose ratios between the three groups, and determined the correlation between plasma glucose levels and CSF glucose, sorbitol and fructose levels.

**Results:**

Groups 2 and 3 had significantly higher CSF fructose levels compared with group 1 (*p*=0.036 and *p*<0.001, respectively). Group 3 showed significant differences compared with groups 1 and 2 for CSF sorbitol (*p*<0.001 and 0.036, respectively). Moreover, patients with diabetes had a significantly higher CSF sorbitol/glucose ratio compared with patients without diabetes. There was a strong positive correlation between plasma glucose and CSF glucose, sorbitol and fructose. Finally, age, sex, CSF/plasma albumin ratio and preoperative cognitive function scores were significantly correlated with plasma glucose and CSF glucose, sorbitol and fructose levels.

**Conclusions/interpretation:**

Hyperglycaemia causes a proportional increase in polyol concentrations in CSF of patients without major neurological disease. Furthermore, this study provides the first indication of upregulation of the cerebral polyol pathway in patients with diabetes without evident neurological abnormalities.

**Graphical abstract:**

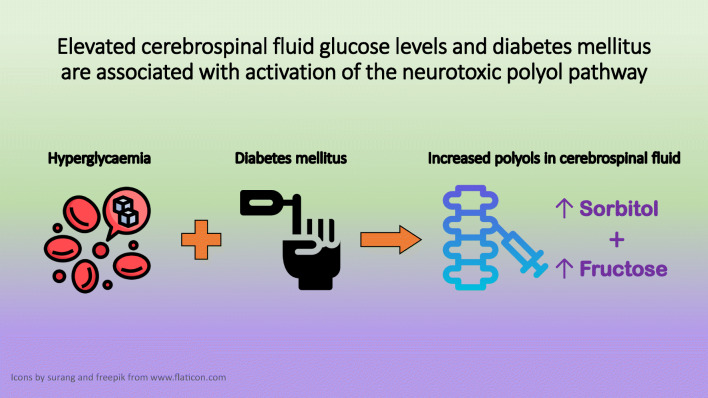

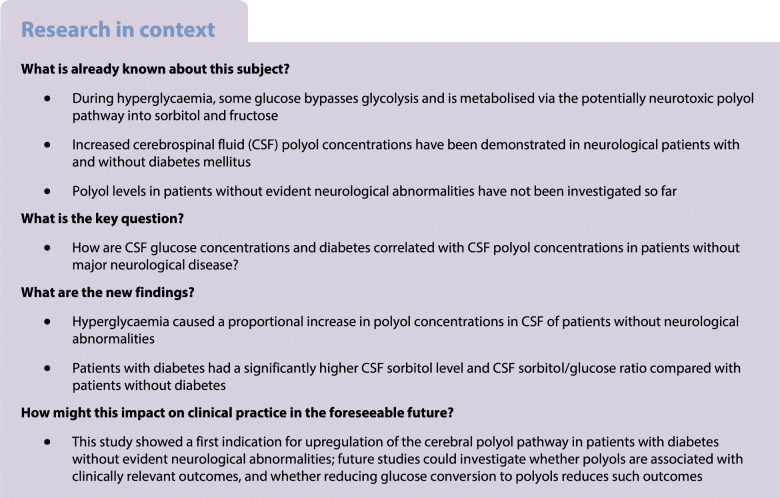



## Introduction

**Fig. 1:**
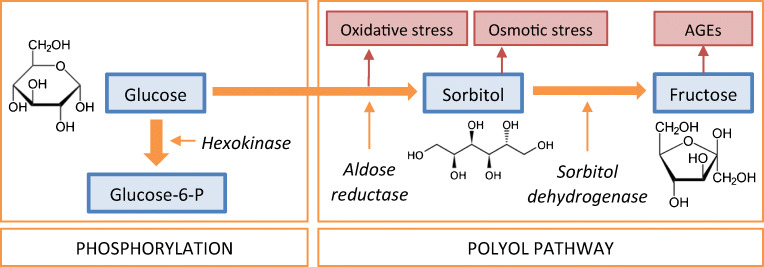
Glucose metabolism and the polyol pathway. Glucose-6-P, glucose 6-phosphate

Under normal physiological conditions, the glycolytic hexokinase pathway metabolises cerebral glucose via brain-specific hexokinase isoforms [1]. However, during hyperglycaemia, some glucose bypasses glycolysis and is isomerised to polyols via the polyol pathway [2], in which aldose reductase first metabolises glucose to the polyol sorbitol, and subsequently sorbitol dehydrogenase oxidises sorbitol to fructose (Fig. 1).

Polyols are cyto- and neurotoxic [3, 4], and excessive polyol concentrations may lead to nephro- [5], retino- [6] and polyneuropathy [7], with sorbitol and fructose playing crucial roles. Sorbitol has an osmotic potential because of limited permeability through cell membranes, and reduces the ability of cells to protect themselves from the influence of reactive oxygen species [3]. Fructose can damage proteins through irreversible glycation and formation of AGEs [4]. Polyols in cerebrospinal fluid (CSF), through the formation of AGEs, non-enzymatic glycation of myelin or oxidative damage/stress, may also play a role in the development of white matter lesions and cognitive impairment [8–10]. Historically, the existence of the polyol pathway may have provided an evolutionary survival advantage, as fructose increases the storage of fat and glycogen, which may later be used to provide energy and water [11]. Fructose also causes sodium retention, and consequently higher blood pressure, which is an advantage in case of dehydration or salt deficiency [11]. However, modern-day non-physiological problems and processes that cause acute or chronic hyperglycaemia, such as diabetes mellitus, surgery [12–14] or critical illness [15], may lead to more deleterious activation of the polyol pathway.

Previous research has demonstrated low CSF polyol concentrations in normoglycaemic healthy individuals [16] and pregnant women [17], and increased CSF polyol levels in patients with neurological disorders, both with and without diabetes [18–20]. Moreover, continuous intravenous glucose infusion induces fructose formation in the brain during periods of hyperglycaemia [21].

Given the likelihood of saturation of the hexokinase pathway under supraphysiological glucose concentrations, we hypothesised that hyperglycaemia causes a proportional increase in CSF glucose, sorbitol and fructose concentrations, and in ratios of CSF fructose and sorbitol to CSF glucose. However, polyol concentrations and CSF polyol/glucose ratios under varying glycaemic conditions in patients without evident neurological abnormalities have not been investigated to date. Therefore, we aimed to study CSF fructose and sorbitol concentrations and ratios of CSF fructose and sorbitol to CSF glucose in patient groups with either normal or elevated CSF glucose concentrations.

## Methods

### Study design and ethics

For this study**,** we used biomaterial and data from patients enrolled in a prospective cohort study, the Anaesthetic Biobank of Cerebrospinal Fluid (ABC) study. Plasma, CSF and clinical data were obtained from a subset of participants of the ABC repository, which was designed to collect and store CSF from patients without major neurological disease for future neuroscientific research. Patients aged 18 years or older, scheduled for elective surgery under spinal anaesthesia**,** were eligible for inclusion. More extensive details of the study design, patient inclusion, and data and biomaterial collection are described elsewhere [22]. The ABC study was approved by the Medical Ethical Committee of the University Medical Center Groningen (UMCG) (registration number 2016**–**174) and written informed consent was obtained from all participants. The study was performed according to the Declaration of Helsinki as revised in 2013, and the trial was registered with trialregister.nl (number NL9356). All participants signed an informed consent form. Performance, recording, data analysis and reporting were conducted according to the Strengthening the Reporting of Observational Studies in Epidemiology (STROBE) **s**tatement for reporting observational studies.

### Study population and inclusion criteria

From the 487 patients enrolled in the ABC study between October 2016 and November 2020, we selected 30 patients for this sub-study. We first excluded, from the ABC study cohort, patients with a CSF erythrocyte count >500 × 10^6^/l (indicating CSF blood contamination [23]), patients with a known neurometabolic disorder (e.g. Parkinson’s disease, multiple sclerosis, Alzheimer’s disease, phenylketonuria, mitochondrial disorders), or those patients with an undetermined plasma or CSF albumin concentration. We then selected three groups of patients on the basis of their CSF glucose concentrations. For group 1 (control group), we selected ten patients at random whose CSF glucose concentration spanned the spectrum of concentrations considered to be normal (2.2–4.4 mmol/l). Among all patients with elevated CSF glucose concentrations (>4.4 mmol/l), we identified those without a diagnosis of diabetes and those with a diagnosis of type 2 diabetes, and ranked each group according to their CSF glucose concentrations. For these two groups, we selected the ten patients with the highest CSF glucose concentrations.

### Material collection and measurements

Spinal puncture and collection of biomaterials were performed according to standardised procedures [22]. CSF (10 ml) was collected into five consecutive 2 ml syringes during the spinal puncture prior to intrathecal local anaesthetic injection. The first 2 ml syringe was used for routine analyses, including albumin, total protein, glucose, leucocyte and erythrocyte count. The remaining CSF was centrifuged (1000 *g*, 10 min, 4°C) and stored at −80°C. Blood (20.5 ml) was collected during intravenous cannulation prior to surgery. Part of the blood (10.5 ml) was used for routine analyses (albumin, total protein, glucose and leucocyte count); the remaining blood was centrifuged (2000 *g*, 10 min, 4°C) and stored at −80°C for future analysis.

CSF sorbitol and fructose concentrations were measured by the Core Facility Metabolomics of the Amsterdam University Medical Center (Amsterdam UMC) in 300 μl CSF from the second 2 ml fraction by GC/flame ionisation detector, as previously described for plasma [24]. The interassay coefficient of variation was 5.7% for sorbitol and 6.1% for fructose. The limit of detection and limit of quantification were 5 μmol/l for both compounds. First, a test sample from four patients was analysed for plasma and CSF sorbitol and fructose to assess the validity of the laboratory analysis. Second, sorbitol and fructose concentrations in CSF were measured in all patients.

Creatinine assays (as indicator of renal function) were later performed on an aliquot of stored plasma by the Department of Clinical Chemistry at Uppsala University, Sweden.

We obtained relevant demographic data, medication use and medical history from patient records. As part of the ABC study procedures, the Montreal Cognitive Assessment (MoCA), a brief cognitive screening tool for mild cognitive impairment, was used to assess cognitive function preoperatively, if logistically possible.

### Outcome

First, between-group differences in baseline- and intraoperative characteristics and routine laboratory analyses (e.g. albumin and creatinine) were determined. As primary outcome measures, we determined the concentrations of plasma glucose, CSF glucose, sorbitol and fructose for all groups, and calculated the CSF polyol/glucose ratios (CSF sorbitol/CSF glucose and CSF fructose/CSF glucose). We then determined between-group differences in plasma glucose, CSF glucose, sorbitol, fructose, and CSF polyol/glucose ratios for groups 1, 2 and 3. The secondary outcomes were the correlations between plasma glucose levels and CSF glucose, sorbitol and fructose levels, as well as between cognitive function and CSF glucose, sorbitol and fructose. In addition, the correlation between the CSF/plasma quotient of albumin (*Q*_alb_ ratio) and plasma glucose, CSF glucose, CSF sorbitol and CSF fructose was analysed. The *Q*_alb_ is an indicator of blood–CSF barrier permeability, and was calculated by dividing the concentration of albumin in CSF by the concentration of albumin in blood, × 1000. Last, age was correlated with plasma glucose, CSF glucose, CSF sorbitol and CSF fructose, and with CSF sorbitol/glucose and fructose/glucose ratios, and we also determined whether differences existed between men and women for these analytes.

### Sample size

We based our sample size calculation on the expected between-group difference in the plasma concentration of sorbitol. Hwang et al [17] reported a mean plasma sorbitol concentration of 0.17 with an SD of 0.02 in a study of 25 pregnant women with and without diabetes. To detect a between-group difference of 0.03 with an SD of 0.02 using a two-sided α of 0.025 (to correct for two comparisons in three groups), it is necessary to include at least ten patients in each group. Therefore, we included a total of 30 patients in this study.

### Statistical analysis

Analyses were performed using SPSS statistics software (version 23.0; IBM NY, USA). Data are expressed as mean ± SD and as median (IQR), when appropriate, plus range (minimum value – maximum value). We assessed for normality by visual inspection of the frequency distribution, the P–P plot and Q–Q plot, and used the Shapiro–Wilk test when in doubt. Discrete data are displayed by category frequencies and percentages. Either a Pearson or Spearman rank correlation was performed when examining the relationship between two continuous variables. For comparisons between groups, the Mann–Whitney *U* test, Fisher’s exact test, the χ^2^ test, the Fisher−Freeman−Halton test or the Kruskal−Wallis *H* test were used when appropriate. If the Kruskal−Wallis *H* test was significant, Dunn’s pairwise tests were performed for the three pairs of groups. For pairwise comparisons between groups, significance values were adjusted by the Bonferroni correction for multiple tests. A *p* value <0.05 (for a two-tailed test) was considered statistically significant.

## Results

### Study cohort and patient characteristics

Patient characteristics are shown in Table 1. The median age of the total group was 61 years (range 20–78) and 53% were female. Age differed significantly between the three study groups (significant between groups 1 and 2 [*p*=0.012] and groups 1 and 3 [*p*=0.002]), and sex differed significantly between groups 1 and 3 (*p*=0.015). BMI ranged from 19 to 40 kg/m^2^, with a median of 30 for the total sample. There was a significant difference in the American Society of Anesthesiology (ASA) physical status [25] between the groups. MoCA test scores ranged from 16 to 30 points (*n* = 23). Only group 1 had a median MoCA score in the normal range (≥26 points, *n* = 10), groups 2 (*n* = 6) and 3 (*n* = 7) had a significantly lower MoCA score (*p*=0.006 and *p*=0.039, respectively). Median *Q*_alb_ values differed significantly between groups 1 and 3 (*p*<0.001), but were within the normal range in all groups (Table 1). Finally, plasma creatinine was not different between groups (data not shown).

**Table 1:** Patient characteristics

Characteristic	All patients (*n* = 30)	Group 1 (*n* = 10)	Group 2 (*n* = 10)	Group 3 (*n* = 10)	*p* value
Sex, female (%)	16 (53)	9 (90)	5 (50)	2 (20)	0.009**
Age (years)	61 [53–70]; 20–78	51 [25–55]; 20–67	65 [57–75]; 46–78	68 [63–73]; 58–78	0.001**
BMI (kg/m^2^)	30 [25–34]; 19–40	29 [23–32]; 20–34	32 [23–37]; 19–40	32 [27–37]; 21–40	0.345
ASA classification (%)					0.003**
ASA I	8 (27)	7 (70)	1 (10)	0 (0)	
ASA II	17 (57)	3 (30)	6 (60)	8 (80)	
ASA III	5 (17)	0 (0)	3 (30)	2 (20)	
Lifestyle factors					
Smoking (*n* = 28) (%)					0.833
Never	15 (53)	6 (67)	5 (56)	4 (40)	
Current	3 (11)	1 (11)	1 (11)	1 (10)	
Former	10 (36)	2 (22)	3 (33)	5 (50)	
Alcohol use, yes (*n* = 26) (%)	17 (65)	6 (75) (*n* = 8)	4 (50) (*n* = 8)	7 (70) (*n* = 10)	0.669
Surgery type (%)					
Orthopaedic	20 (67)	6 (60)	6 (60)	8 (80)	
Urological	4 (13)	0 (0)	4 (40)	0 (0)	
Surgical	4 (13)	2 (20)	0 (0)	2 (20)	
Gynaecological	2 (7)	2 (20)	0 (0)	0 (0)	
MoCA score (*n* = 23)	27 [23–29]; 16–30	29 [28–30]; 26–30 (*n* = 10)	24 [18–26]; 18–27 (*n* = 6)	25 [21–28]; 16–30 (*n* = 7)	0.003**
*Q*_alb_ (× 10^−3^)	5.3 [4.1–7.6]; 2.0–15.2	4.1 [3.3–4.6]; 2.0–5.4	4.9 [4.2–5.8]; 3.8–10.6	8.1 [6.1–10.6]; 4.4–15.2	<0.001***
Plasma creatinine (μmol/l) (*n* = 29)	71 [60–88]; 39–132	69 [59–77]; 54–87	80 [59–116]; 39–132 (*n* = 9)	77 [59–95]; 58–129	0.464

Data are median [IQR] with range, or frequency (%)

Group 1, control group; group 2, elevated CSF glucose; group 3, type 2 diabetes

**p<0.01; ***p<0.001 level (two-tailed)

Of the 30 patients included, 93% had at least one comorbid condition (other than the reason for surgery), and 90% were using at least one medication. Twenty-six of the patients had not suffered from an active central nervous system disorder in the 12 months preceding the collection of biomaterials. The four patients who did have a central nervous system disorder in the 12 months before biomaterial collection included one patient from group 1 (depression), two patients from group 2 (epilepsy; small right thalamic infarction >6 months prior with no current medication use), and one patient from group 3 (facial nerve schwannoma). One patient from group 1, three patients from group 2, and no patients from group 3 used corticosteroids. All patients in group 3 used glucose-lowering medication. Five patients were on insulin therapy, of whom three used a combination of rapid-acting and long-acting insulin, one patient used rapid-acting and intermediate-acting insulin, and one patient used rapid-acting insulin together with ultra-long-acting insulin. Four patients were on oral glucose-lowering drugs, including biguanides, sulfonylurea derivatives and dipeptidyl peptidase 4 inhibitors. One patient used a combination of rapid-acting and long-acting insulin and a biguanide.

### Primary outcome

**Fig. 2:**
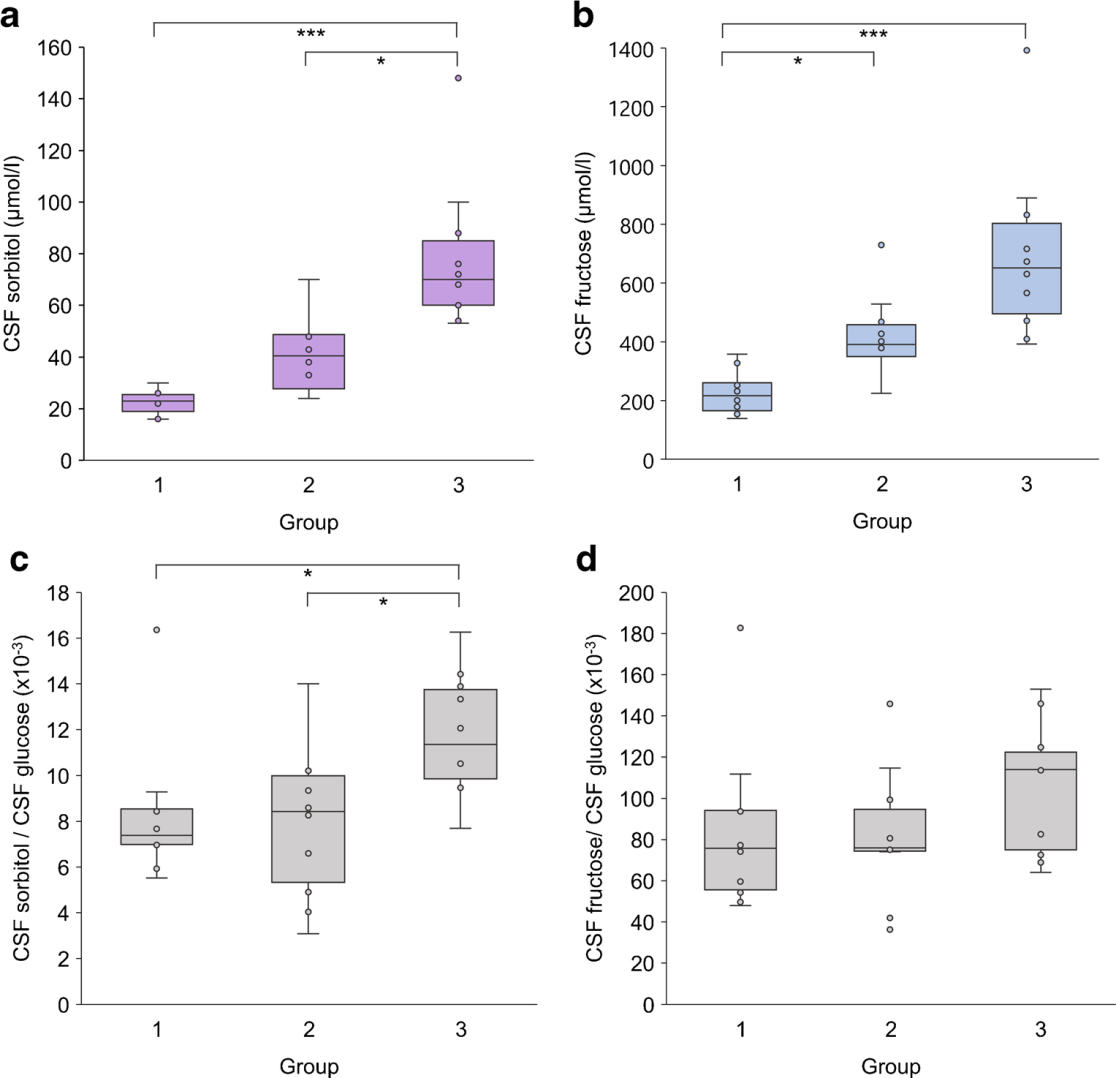
Cerebrospinal fluid (CSF) concentrations of polyols. Concentrations of CSF sorbitol (**a**), concentrations of CSF fructose (**b**), CSF sorbitol/CSF glucose ratio (**c**) and CSF fructose/CSF glucose ratio (**d**) for group 1 (control) (*n* = 10), group 2 (elevated CSF glucose) (*n* = 10) and group 3 (diabetes mellitus) (*n* = 10). *p<0.05, **p<0.01 and ***p<0.001

Plasma glucose and CSF glucose, sorbitol and fructose values are shown in Table 2. There was a broad range for plasma glucose concentrations (4.4–16.8 mmol/l) and CSF glucose concentrations (1.1–9.1 mmol/l) when all patients were grouped together. A significant difference was observed between the three groups for plasma glucose and CSF glucose, sorbitol and fructose (all *p*<0.001). Pairwise comparisons between groups showed significant differences for plasma glucose levels between groups 1 and 2 (*p*=0.030) and groups 1 and 3 (*p*<0.001). CSF glucose concentrations differed significantly between groups 1 and 2 (*p*=0.009) and groups 1 and 3 (*p*<0.001). Group 3 showed a significant difference compared with group 1 for both CSF sorbitol (*p*<0.001) and fructose levels (*p*<0.001), and also showed a significant difference for CSF sorbitol compared with group 2 (*p*=0.036) (Fig. 2). CSF fructose concentrations also differed between groups 1 and 2 (*p*=0.036).

**Table 2:** Results of biochemical analyses of CSF and plasma

	All patients (*n* = 30)	Group 1 (*n* = 10)	Group 2 (*n* = 10)	Group 3 (*n* = 10)	*p* value
Plasma glucose (mmol/l) (*n* = 29)	7.6 [5.5–10.9]; 4.4–16.8	5.1 [4.9–5.5]; 4.4–5.7 (*n* = 9)	7.7 [6.4–8.2]; 5.5–11.1	11.7 [10.0–14.2]; 7.0–16.8	<0.001***
CSF glucose (mmol/l)	5.0 [3.3–6.1]; 1.1–9.1	3.1 [2.8–3.3]; 1.1–3.5	5.0 [4.7–5.8]; 4.6–7.8	6.2 [5.7–7.4]; 5.4–9.1	<0.001***
CSF sorbitol (μmol/l)	41 [24–62]; 16–148	23 [18–26]; 16–30	41 [26–49]; 24–70	70 [53–91]; 53–148	<0.001***
CSF fructose (μmol/l)	387 [247–582]; 139–1392	217 [159–280]; 139–358	391 [338–482]; 225–729	652 [456–847]; 393–1392	<0.001***
CSF sorbitol/CSF glucose (× 10^−3^)	8.9 [7.0–11.0]; 3.1–16.4	7.4 [6.7–8.8]; 5.5–16.4 (*n* = 9)	8.4 [4.7–10.3]; 3.1–14.0	11.3 [9.6–14.0]; 7.7–16.3	0.009**
CSF fructose / CSF glucose (× 10^−3^)	79 [68–115]; 36–183	75 [53–99]; 48–183	76 [66–103]; 36–146	114 [72–130]; 64–153	0.224

Data are median [IQR] with range, or frequency (%)

Group 1, control group; group 2, elevated CSF glucose; group 3, type 2 diabetes

**p<0.01; ***p<0.001 level (two-tailed)

Our test samples showed that the detection limit of sorbitol and fructose in plasma and CSF was 6 μmol/l when processing 500 μl of material. Sorbitol was not detectable in plasma, and plasma fructose showed an abnormal peak pattern compared with what would be expected for CSF fructose (data not shown). Therefore, it was decided not to determine plasma polyols for the entire study population.

### Secondary outcomes

**Fig. 3:**
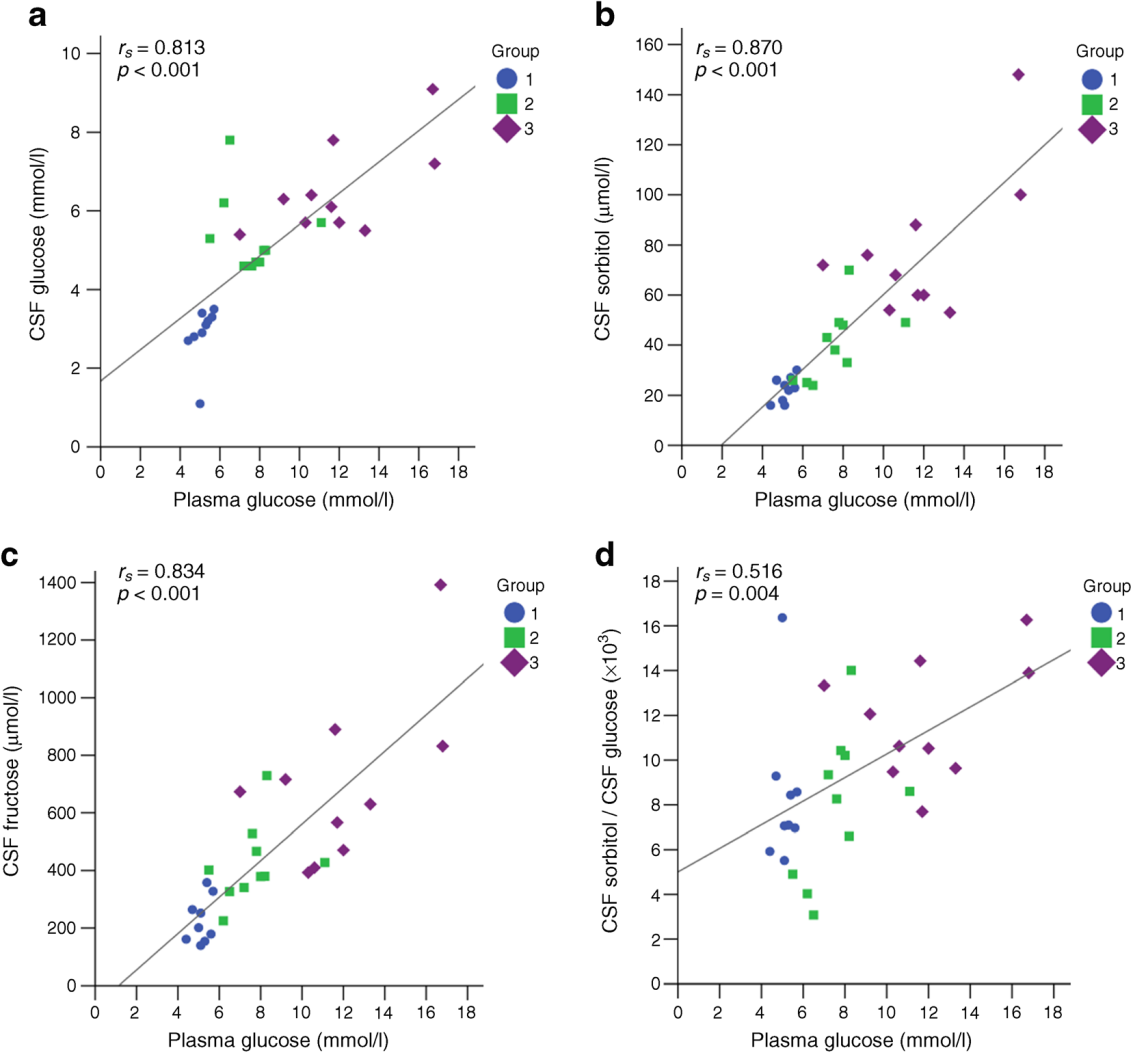
Correlations between plasma glucose and (**a**) CSF glucose, (**b**) CSF sorbitol, (**c**) CSF fructose and (**d**) CSF sorbitol/CSF glucose ratio (total *n* = 30). Group 1, control group; Group 2, elevated CSF glucose; Group 3, type 2 diabetes

The correlations between plasma and CSF glucose, sorbitol and fructose, MoCA scores, *Q*_alb_ and age are shown in Table 3. There was a strong positive correlation between plasma glucose and CSF glucose (*r*_s_ = 0.813, *p*<0.001), sorbitol (*r*_s_ = 0.870, *p*<0.001) and fructose (*r*_s_ = 0.834, *p*<0.001) when all patients were grouped together (Table 3 and Fig. 3a–c). Moreover, CSF sorbitol correlated strongly with CSF fructose in the total study group (*r*_s_ = 0.945, *p*<0.001), as well as in group 1 (*r*_s_ = 0.909, *p*<0.001), group 2 (*r*_s_ = 0.705, *p*=0.023) and group 3 (*r*_s_ = 0.827, *p*=0.003) individually.

**Table 3:** Correlations for the polyol pathway

Total group (*n* = 30)	Plasma glucose	CSF glucose	CSF sorbitol	CSF fructose
Plasma glucose	NA	0.813*** (*n* = 29)	0.870*** (*n* = 29)	0.834*** (*n* = 29)
CSF glucose	NA	NA	0.748**	0.705***
CSF sorbitol	NA	NA	NA	0.945***
CSF fructose	NA	NA	NA	NA
MoCA (*n* = 23)	−0.599** (*n* = 22)	−0.553**	−0.512*	−0.579**
*Q*_alb_	0.664*** (*n* = 29)	0.709***	0.637***	0.606***
Age	0.725*** (*n* = 29)	0.633***	0.639***	0.593**

*p<0.05; **p<0.01; ***p<0.001 level (two-tailed)

NA, not applicable

Preoperative MoCA scores were moderately inversely correlated with plasma glucose (*p*<0.01), CSF glucose (*p*<0.01), CSF sorbitol (*p*<0.05) and CSF fructose (*p*<0.01) (Table 3). There was a moderate positive correlation between *Q*_alb_ and plasma glucose, CSF glucose, CSF sorbitol and CSF fructose (all *p*<0.001).

In all patients grouped together, plasma glucose correlated with the CSF sorbitol/glucose ratio (*r*_s_ = 0.516, *p*=0.004) (Fig. 3d), but not with the CSF fructose/o-glucose ratio. CSF glucose was strongly significantly correlated with CSF sorbitol (*r*_s_ = 0.748, *p*<0.001) and CSF fructose (*r*_s_ = 0.705, *p*<0.001). The CSF sorbitol/glucose ratio differed significantly across the groups (*p*=0.009), and there was a significant difference in the ratio between groups 1 and 3 (*p*=0.017) and between groups 2 and 3 (*p*=0.031) (Fig. 2c). The CSF fructose/glucose ratio did not differ between the groups (*p*=0.224) (Fig. 2d).

Age was positively correlated with plasma glucose and CSF glucose, sorbitol and fructose (all *p*≤0.001) (Table 3), and the CSF sorbitol/glucose ratio (*p*=0.015), but not with the CSF fructose/glucose ratio (*p*=0.188). Finally, there was a significant difference between men and women for plasma glucose, CSF glucose and CSF sorbitol/glucose ratio (all *p*<0.05), and for CSF sorbitol and CSF fructose (*p*<0.01), but not for the CSF fructose/glucose ratio (*p*=0.146).

## Discussion

In this study, patients with elevated CSF glucose levels also had significantly higher CSF sorbitol and fructose levels. Even when CSF glucose values were similar, patients with diabetes had a significantly higher CSF fructose, CSF sorbitol and CSF sorbitol/glucose ratio compared with patients without diabetes. Furthermore, CSF glucose, sorbitol and fructose concentrations increased linearly with an increase in systemic glucose levels. These findings are in line with our primary hypothesis that hyperglycaemia causes a proportional increase in polyol concentrations in CSF of patients without brain disorders.

This proof-of-concept study is relevant because we demonstrate an activation of the polyol pathway across a broad spectrum of plasma and CSF glucose levels in patients both with and without diabetes. Activation of the polyol pathway is associated with possible cyto- and neurotoxic properties, and is thought to play a role in brain abnormalities and cognitive impairment through the formation of AGEs, non-enzymatic glycation of myelin or oxidative damage/stress [8–10]. For example, MRI has shown that a higher percentage of white matter hyperintensities are found in patients with affective disorders, and that these white matter hyperintensities are associated with increased glucose metabolism of the brain via the polyol pathway [8]. Furthermore, hyperglycaemia is associated with structural neuronal changes and impaired long-term spatial memory [26].

Our findings are consistent with the principle of Michaelis–Menten kinetics [27], which states that an increase in systemic glucose proportionally increases cerebral glucose via facilitated transport [27]. In addition, sorbitol and fructose concentrations in the CSF were comparable to those found in studies of patients with normal systemic glucose values [16, 17]. Activation of the polyol pathway during hyperglycaemia was demonstrated in the 1970s in patients with diabetes, but these patients were hospitalised for neurological disorders and thus were unlikely to have an intact blood–CSF barrier [18–20].

Interestingly, the CSF polyol/glucose ratios were exponentially increased when patients had diabetes, despite the fact that the CSF glucose levels of groups 2 and 3 were similar and there were no between-group differences in age, sex, MoCA scores or *Q*_alb_. Thus, the differences between the two groups could not be attributed to these factors. The steep increase may be due to a statistical dispersion; however, no such correlation was demonstrated between CSF sorbitol/systemic glucose ratios and between CSF fructose/systemic glucose ratios. Possibly it is due to upregulation of the cerebral polyol pathway in diabetes patients because of chronic hyperglycaemia, combined with a saturation of the hexokinase pathway, leaving more glucose in the CSF for the polyol pathway. It has previously been shown that a state of chronic hyperglycaemia leads to an upregulation of the polyol pathway [28, 29], and, although this upregulation has not yet been demonstrated in the human brain [17], there is previous evidence for elevated glucose, sorbitol and fructose levels in the brains of a rat model for diabetes [30].

Plasma glucose correlated significantly with the CSF sorbitol/glucose ratio, but not with the CSF fructose/glucose ratio. Possibly this difference may be related to fructose catabolism by redirection of fructose to the pentose phosphate pathway via hexokinase or ketohexokinase [31]. Alternatively, this finding may be explained by the formation of precursors of AGEs such as 3-deoxyglucosone or methylglyoxal through non-enzymatic glycation of fructose. From fructose, the enzyme phosphokinase produces fructose-3-phosphate. Both fructose and fructose-3-phosphate may be converted to 3-deoxyglucosone. In addition, fructose may be converted into triose phosphate by the action of fructokinase [32].

The measurement of polyols in CSF is a derivative measure of cerebral metabolism. As polyols can also be transported across the blood–CSF barrier, one may question the validity of CSF polyol values as a marker of cerebral glucose metabolism [33]. However, the measured plasma polyol concentrations were markedly lower than in the CSF or even below the limit of quantification. It has been previously demonstrated that the human brain produces polyols [21], and that the primary source of polyols in CSF is from the brain itself [21]. The higher levels of sorbitol and fructose in the CSF compared with plasma in our cohort also indicate this. Furthermore, the glucose, sorbitol and fructose CSF concentrations in our cohort correspond to those measured in previous studies [16–20]. Thus, it appears that changes in CSF sorbitol and fructose concentrations do indeed reflect changes in cerebral glucose metabolism.

A limitation of the present study is that the data do not provide information about changes in glucose, sorbitol and fructose concentrations in the CSF over time, as these could not be determined given our current study design. Proof of activation of the cerebral polyol pathway over time could be provided by infusion of stable isotope-labelled glucose and subsequent recovery of these isotope labels in the sorbitol and fructose. In addition, a relatively small sample size was used, which did not allow statistical correction for patient characteristics such as age, sex, ASA physical status classification or MoCA scores. This may be remedied in the future by using a larger sample size. Finally, it is possible that some patients in group 2 may have had undiagnosed type 2 diabetes, and not all data important for diabetes patients were known (e.g. lipid status and HbA_1c_ levels); it would be preferable to include these diagnostic measurements in future studies of diabetes patients.

It is important to study whether these harmful metabolites are related to patient-relevant postoperative complications, and our results show that future studies can explore the potential of systemic glucose-lowering therapies such as insulin or incretin-based therapies to optimise cerebral glucose and polyol metabolism [34]. Suppression of the cerebral polyol pathway may, in theory, reduce cognitive impairment, and the role of (elevated) polyol concentrations in the development of cognitive decline should be investigated.

In conclusion, this study has demonstrated that, even in patients without evident neurological abnormalities, glucose is converted to polyols in the polyol pathway. We have shown increased levels of cerebral polyols in patients with diabetes, which is a first indication of an upregulation of the cerebral polyol pathway in patients with diabetes without evident neurological abnormalities. Future studies could further investigate how this increase in polyols in the CSF evolves over time, and whether they are indeed associated with clinically relevant patient outcomes, and if so, whether reducing glucose conversion in the polyol pathway results in a reduction in the incidence of such outcomes.

## Data Availability

De-identified data are available upon reasonable request.

## Abbreviations

ABC: Anaesthetic Biobank of Cerebrospinal Fluid
ASA: American Society of Anesthesiology
CSF: Cerebrospinal fluid
MoCA: Montreal Cognitive Assessment
*Q*_alb_: CSF/plasma quotient of albumin
